# Induction of LTP mechanisms in dually innervated dendritic spines

**DOI:** 10.1038/s41598-024-66871-8

**Published:** 2024-07-09

**Authors:** Jonathan E. Tullis, K. Ulrich Bayer

**Affiliations:** 1 Department of Pharmacology, University of Colorado Anschutz Medical Campus, Aurora, CO 80045 USA; 2 Program in Neuroscience, University of Colorado Anschutz Medical Campus, Aurora, CO 80045 USA

**Keywords:** Cellular neuroscience, Molecular neuroscience, Synaptic plasticity

## Abstract

Dendritic spines are the postsynaptic compartments of excitatory synapses, however, a substantial subset of spines additionally receives inhibitory input. In such dually innervated spines (DiSs), excitatory long-term potentiation (LTP) mechanisms are suppressed, but can be enabled by blocking tonic inhibitory GABA_B_ receptor signaling. Here we show that LTP mechanisms at DiSs are also enabled by two other excitatory LTP stimuli. In hippocampal neurons, these chemical LTP (cLTP) stimuli induced robust movement of the Ca^2+^/calmodulin-dependent protein kinase II (CaMKII) to DiSs. Such synaptic CaMKII accumulation is an essential LTP mechanism at singly innervated spines (SiSs). Indeed, CaMKII accumulation at DiSs was also accompanied by other readouts for successful LTP induction: spine growth and surface insertion of GluA1. Thus, DiSs are capable of the same LTP mechanisms as SiSs, although induction of these mechanism additionally requires either reduced inhibitory signaling or increased excitatory stimulation. This additional regulation may provide further computational control.

## Introduction

Neuronal computation that underlies higher brain functions requires excitatory synapses (that use excitatory transmitters such as glutamate), inhibitory synapses (that use inhibitory transmitters such as γ-aminobutyric acid (GABA)), and the regulatory cross-talk between both synapse types^1,2^. Excitatory synapses form typically on dendritic spines, whereas inhibitory synapses typically form directly onto the dendritic shaft. However, a substantial portion of the spines in the neocortex and hippocampus additionally receive inhibitory input. These dually innervated spines (DiSs) account for ~ 5–30% of all excitatory and inhibitory synapses, respectively^3–5^. Compared to excitatory singly innervated spines (SiSs) and to the singly innervated inhibitory synapses that form on dendritic shafts (I-Syns), these DiSs have the potential to provide additional mechanisms for signal computation, although very little is known about this to date. One form of synaptic signal computation that is thought to be important for learning and memory is long-term potentiation (LTP) at hippocampal excitatory synapses^6,7^. LTP induction is well established to require the Ca^2+^/calmodulin-dependent protein kinase II (CaMKII)^8–10^, and we recently showed that this requires structural rather than enzymatic CaMKII functions^11^. Specifically, these structural functions involve the regulated binding of CaMKII to the NMDA-type glutamate receptor (NMDAR) subunit GluN2B, an interaction that mediates the further accumulation of CaMKII at excitatory synapses after LTP stimuli^12–16^. Interestingly, a recent study showed that LTP is suppressed at DiSs in hippocampal neurons^3^, raising the possibility that this is caused by suppression of CaMKII movement. Additionally, this study showed that LTP mechanisms at DiSs could be successfully induced when tonic inhibitory GABA_B_ receptor (GABA_B_R) signaling was blocked^3^. Here we show that stronger excitatory stimuli can also lead to successful induction of LTP mechanisms at DiSs. The LTP mechanisms observed at DiSs included CaMKII accumulation, spine growth, and surface insertion of the AMPA-type glutamate receptor (AMPAR) subunit GluA1. Thus, DiSs are intrinsically capable of the same LTP mechanisms described in SiSs, but these mechanisms may differ in the stimulation threshold that is required for their induction at DiSs versus SiSs.

## Results

### Identification of dually-innervated spines (DiSs)

**Figure 1:**
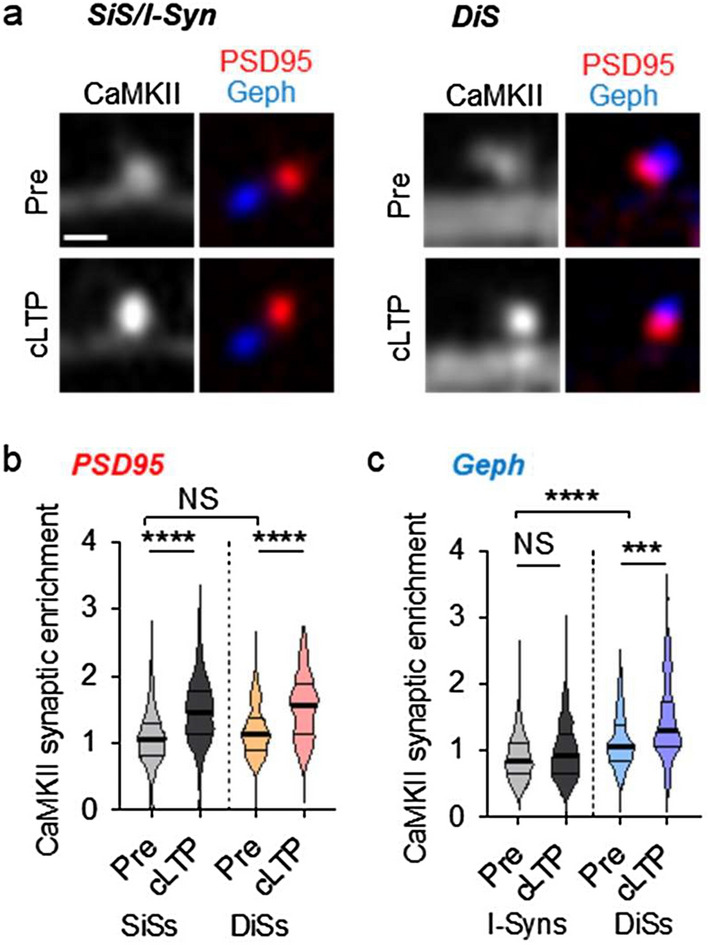
CaMKII accumulation at DiSs after cLTP by glutamate bath application. Representative images of hippocampal neurons and quantification of CaMKII localization illustrated by violin plots (thick line: median; thin lines: interquartile range). (**a**) Representative images of CaMKII (grey; intrabody labelled with YFP2), PSD95 (red; intrabody labelled with mCherry), gephyrin (blue; intrabody labelled with mTurqouise) before and 5 min after bath application of glutamate (cLTP); scale bar, 1 µm. The three synapse types shown are SiS (PSD95 alone), I-Syn (gephyrin alone), and DiS (PSD95 and gephyrin within 0.5 µm); the four distinct groups for quantification are PSD95 puncta at SiS versus DiS, and gephyrin puncta at I-Syns versus DiS. (**b**) cLTP increased synaptic enrichment of CaMKII within PSD95 puncta at both SiSs and DiSs; n = 671, 717, 118, 119 synapses from 9 neurons; ****p < 0.0001; Welch’s one-way ANOVA with Dunnett’s multiple comparisons test. (**c**) cLTP increased synaptic enrichment of CaMKII within gephyrin puncta at DiSs but not I-Syns; n = 306, 250, 118, 119 synapses from 9 neurons; ***p < 0.001, ****p < 0.0001; Welch’s one-way ANOVA with Dunnett’s multiple comparisons test.

To identify and analyze distinct populations of dendritic spines, we utilized fluorescently labelled intrabodies for simultaneous live imaging of endogenous CaMKII together with endogenous PSD95 and gephyrin (Fig. 1a), two marker proteins for excitatory and inhibitory synapses, respectively, as previously described^14,17,18^. DiSs were identified by PSD95 puncta with proximal gephyrin labeling within 0.5 µm (measured as center-to-center distance between objects); SiSs were identified by PSD95 puncta that lack such a proximal gephyrin marker within 0.75 µm. PSD95 puncta with gephyrin markers within the intermediate range of 0.5–0.75 µm were excluded from the analysis, as their identity as SiS or DiS is less clear (Fig. 1a and Supplementary Fig. S1a–c). While these cutoff distances are somewhat arbitrary, they are based on the expected spine size. Exclusion of a range of uncertainty further improves accuracy, and visual inspection confirms the chosen cutoff values as reasonable (Fig. 1a and Supplementary Fig. S1a–c). For instance, the example images in Fig. 1a show PSD95 to gephyrin distances of 1.78 µm (SiS) and 0.33 µm (DiS). Conversely, the gephyrin puncta that mark inhibitory synapses reside either isolated as singly-innervated inhibitory synapses (I-Syns) on dendritic shafts or jointly with a PSD95 puncta within a DiS. The I-Syns were classified using the same nearest-neighbor methodology, i.e. as gephyrin puncta that lack any PSD95 marker within a range of 0.75 µm (Fig. 1a and Supplementary Fig. S1a–c).

Based on this classification method, 13.5 ± 1.5% of all dendritic spines on our hippocampal neurons were DiSs; conversely, these DiSs represented 31.7 ± 3.7% of all inhibitory input (Supplementary Fig. S1d). Both of these measures are well aligned with previously published numbers in pyramidal neurons in hippocampus^3^ or cortex^4,19–22^.

### CaMKII is more basally enriched at both SiSs and DiSs compared to I-Syns

As CaMKII is slightly enriched within dendritic spines even under basal conditions^13,23^, we examined whether this CaMKII enrichment differed between SiS vs DiS. For this analysis, CaMKII basal synaptic enrichment was measured within the PSD objects of SiSs versus DiSs. Similarly, CaMKII enrichment was measured within the gephyrin objects of I-Syns versus DiSs. Within the PSD95 objects, basal CaMKII synaptic enrichment did not differ between SiS vs DiS (Fig. 1a,b and Supplementary Fig. S1e). Thus, CaMKII enrichment in spines is seen not only in SiS but also in DiSs. Within gephyrin puncta, CaMKII synaptic enrichment was significantly higher within the DiS group compared to the I-Syns group (Fig. 1a,c and Supplementary Fig. S1f), as expected. This further supports that (i) CaMKII is basally enriched at both SiS and DiS, and (ii) our methods adequately identify the distinct synapse populations.

### CaMKII accumulation at DiSs after cLTP by glutamate bath application

In order to test if CaMKII synaptic accumulation is impaired at DiSs compared to SiSs, we measured CaMKII synaptic enrichment 5 min after a strong glutamate-induced cLTP stimulus (45 s bath application of 100 µM glutamate in the presence of glycine). Such cLTP stimuli have been shown to increase both AMPA-type glutamate receptor surface expression^24,25^ and CaMKII enrichment at SiSs^12–14,26^ in hippocampal neurons. After such cLTP stimuli, CaMKII synaptic enrichment within PSD95 puncta increased significantly, not only in SiSs but also in DiSs (Fig. 1b and Supplementary Fig. S1e). Thus, strong LTP stimuli can trigger CaMKII accumulation also in DiSs.

Additionally, we assessed the cLTP-induced changes of CaMKII accumulation within gephyrin puncta. As expected, we did not observe any increase in CaMKII synaptic enrichment within gephyrin puncta that lacked proximal PSD95 staining (Fig. 1c), confirming that cLTP-stimuli do not cause CaMKII enrichment at I-Syns^17,24^. However, for gephyrin puncta with proximal PSD95 staining (i.e. in DiSs), a significant cLTP-induced increase in CaMKII enrichment was observed (Fig. 1c and Supplementary Fig. S1f). Even though the assumption is that CaMKII accumulates at the excitatory part of the DiS, the presumed difference in the enrichment within their PSD95 versus gephyrin areas was not distinguishable with our method. Nonetheless, this analysis further confirms that CaMKII can accumulate at DiSs in response to strong LTP stimuli.

### CaMKII accumulation at DiSs after cLTP that evokes synaptic stimulation

**Figure 2:**
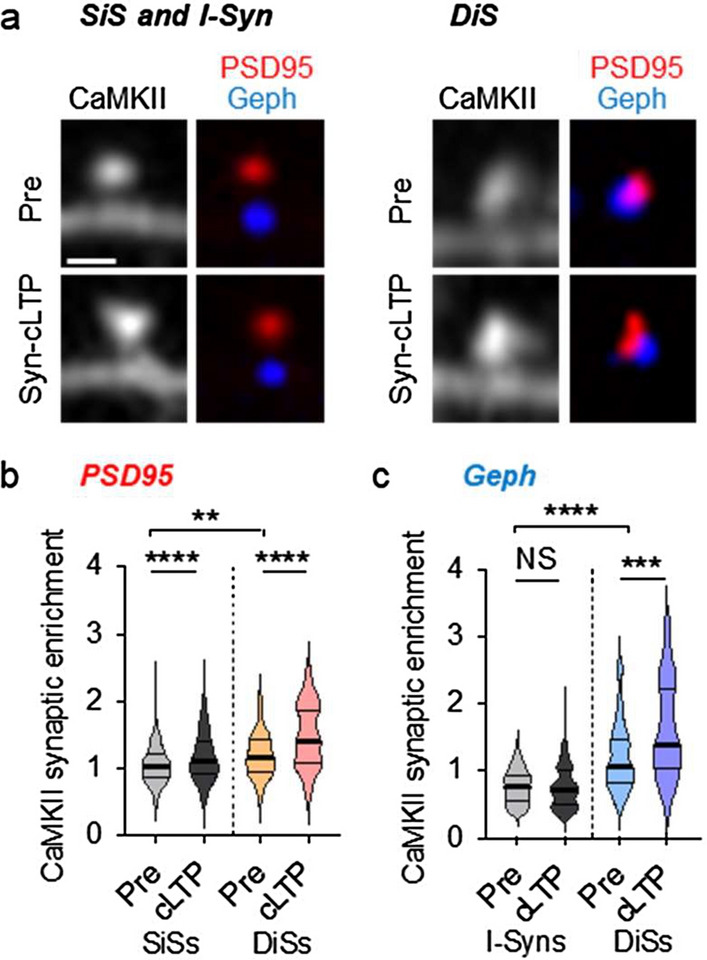
CaMKII accumulation at DiSs after cLTP that evokes synaptic stimulation. Representative images of hippocampal neurons and quantification of CaMKII localization illustrated by violin plots (thick line: median; thin lines: interquartile range). (**a**) Representative images of CaMKII (grey; intrabody labelled with YFP2), PSD95 (red; intrabody labelled with mTurqouise), gephyrin (blue; intrabody labelled with mScarlet) before and 20 min after synaptically-induced cLTP (syn-cLTP); scale bar, 1 µm. (**b**) Syn-cLTP increased synaptic enrichment of CaMKII within PSD95 puncta at both SiSs and DiSs; n = 712, 683, 76, 92 synapses from 7 neurons; **p < 0.01, ****p < 0.0001; Welch’s one-way ANOVA with Dunnett’s multiple comparisons test. (**c**) Syn-cLTP increased synaptic enrichment of CaMKII within gephyrin puncta at DiSs but not I-Syns; n = 148, 144, 76, 93 synapses from 7 neurons; ***p < 0.001, ****p < 0.0001; Welch’s one-way ANOVA with Dunnett’s multiple comparisons test.

We next tested the effects of a different chemical stimulation that is also strong but that elicits excitatory LTP by synaptic release of glutamate release (syn-cLTP). This method involves incubating the cells with TTX (1 µM) for one hour in artificial cerebral spinal fluid (ACSF), then stimulating neurons by removing TTX and magnesium, while adding glycine (200 µM) for 5 min. This strategy for syn-cLTP is well established^27–29^ and relies on the combination of enhancing presynaptic glutamate release (by TTX-withdrawal) and increasing the postsynaptic NMDAR responses (by removing magnesium and adding glycine). Cells were then returned to standard ACSF containing magnesium to quench the stimulus (Supplementary Fig. S2a). After such syn-cLTP, we again detected CaMKII accumulation within PSD95 puncta at both SiSs and DiSs (Fig. 2a,b and Supplementary Fig. S1b). Within gephyrin puncta, CaMKII accumulated after syn-cLTP only at DiSs (Fig. 2a,c and Supplementary Fig. S1c), as expected. No change in synapse size was observed for either PSD95 or gephyrin puncta (Supplementary Fig. S2d,e). In contrast to the syn-cLTP treatments, when cells were treated with ACSF control buffer, no CaMKII accumulation was detected in either dendritic spine type or inhibitory synapse type (Supplementary Fig. S2f–h).

Note that in addition to the further accumulation of CaMKII seen at both SiSs and DiSs in response to the syn-cLTP stimuli, there was also an apparently higher “basal” CaMKII enrichment in DiSs compared to SiSs (see Fig. 2b), which was not seen in the basal conditions of our other cLTP stimulus (see Fig. 1b). In contrast to our other cLTP stimulus, the syn-cLTP stimulus is preceded by a 60 min treatment with TTX, suggesting that the basal synaptic CaMKII enrichment could have a higher sensitivity to TTX at SiSs compared to DiSs.

### Structural and functional LTP at DiSs measured by spine size and GluA1 insertion

Our experiments indicated that strong cLTP stimuli can induce at least one LTP mechanism in DiSs: accumulation of CaMKII. Thus, we decided to test if strong cLTP stimuli can also induce more directly LTP-associated mechanisms in DiSs: spine growth and surface insertion of AMPA-type glutamate receptors. Changes in spine size were measured using Halotag-647 as a cell fill. In response to syn-cLTP, significant spine growth was observed for both SiSs and DiSs (Supplementary Fig. S3a–d). Although the spine growth appeared slightly more extensive for SiSs, this was not statistically significant (Supplementary Fig S3b–d).

**Figure 3:**
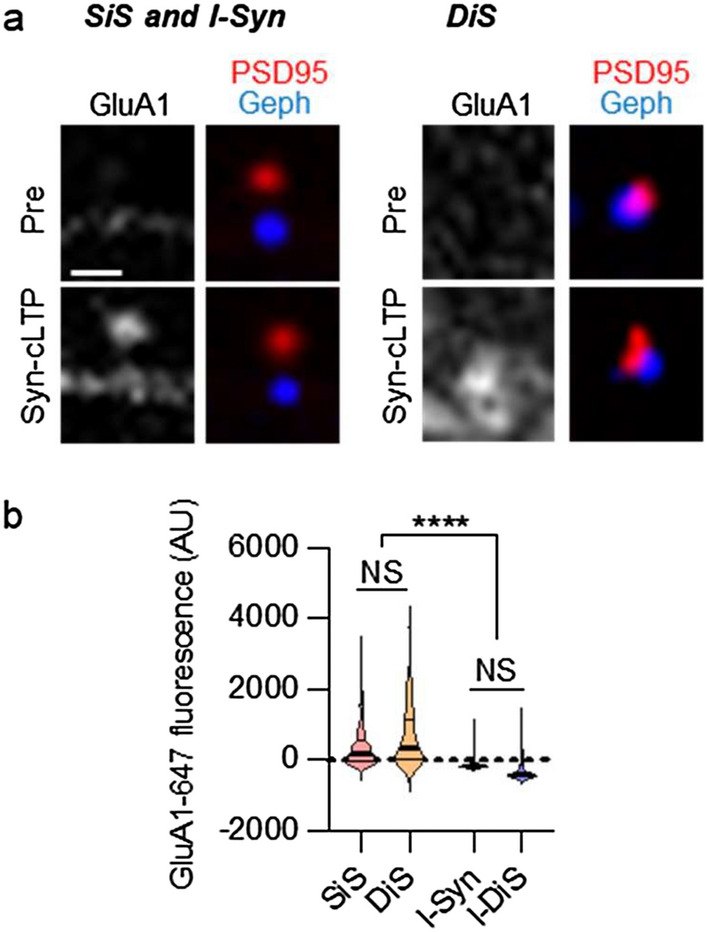
Functional LTP at DiSs measured by GluA1 surface insertion. Representative images of hippocampal neurons and quantification of GluA1 surface expression illustrated by violin plots (thick line: median; thin lines: interquartile range). This was done in the same neurons that were analyzed for CaMKII movement in Fig. 2. (**a**) Representative images of GluA1-647 antibody labelling after syn-cLTP; scale bar, 1 µm. (**b**) Syn-cLTP increased GluA1 surface expression within PSD95 puncta at SiSs and DiSs (measured after blocking the pre-existing surface GluA1 with unlabeled antibody), but significantly less within gephyrin puncta at either SiISs or DiSs; n = 683, 92, 144, 93 synapses from 7 neurons; ****p < 0.0001; Welch’s one-way ANOVA with Dunnett’s multiple comparisons test.

Additionally, we measured the syn-cLTP-induced surface insertion of GluA1. This was done on the same data as set shown in Fig. 2 for CaMKII movement. The timeline of the method is illustrated in Supplementary Fig. S2a. Briefly, and similar as described before^25,28,30^, unlabelled GluA1 antibody was used to block existing surface GluA1 for 1 h during the TTX silencing phase, then washed out prior to initiating syn-cLTP. After the 5 min syn-LTP stimulus, Alexa-647-labelled GluA1 antibody was added to detect newly inserted GluA1 that was not blocked by the first antibody incubation. Excess antibody was washed out after 20 min and the neurons were imaged. The GluA1 signal was measured within PSD95 and gephyrin puncta and stratified for subpopulation analysis. Significantly higher GluA1 surface insertion was detected within PSD95 puncta compared to gephyrin puncta (Fig. 3a,b and Supplementary Fig. S3d). Within the PSD95 and gephyrin puncta, there was no difference in the GluA1 signal for SiSs versus DiSs, or I-Syns versus DiSs, respectively (Fig. 3a,b). No GluA1 accumulation was detected in ACSF-treated neurons (Supplementary Fig. S4a,b). This indicates that the surface expression of newly-inserted GluA1 was localized explicitly to the excitatory component of DiSs after syn-cLTP, as expected.

### GluA1 insertion correlates with CaMKII accumulation at both SiSs and DiSs

**Figure 4:**
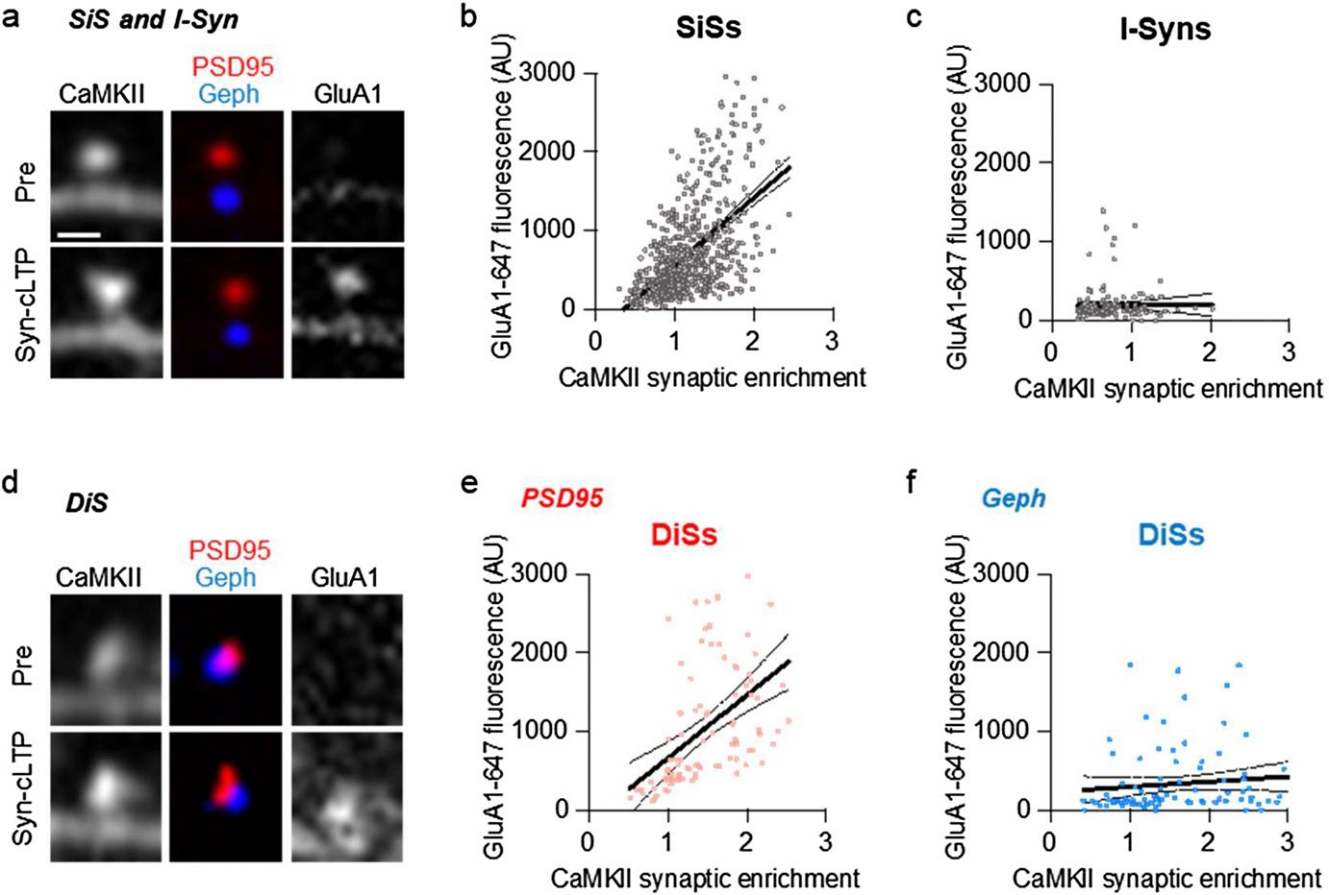
GluA1 insertion correlates with CaMKII accumulation at both SiSs and DiSs. Syn-cLTP-induced GluA1 surface expression was significantly correlated with CaMKII synaptic enrichment at PSD95 puncta but not at gephyrin puncta. This correlation was enabled as the same neurons were used for measuring CaMKII synaptic enrichment (Fig. 2) and GluA1 surface insertion (Fig. 3). (**a**) Representative images of CaMKII enrichment and GluA1 labelling at SiS, I-Syn, and DiS before and after syn-cLTP; scale bar, 1 µm. (**b**) Correlation at SiSs; n = 683 synapses from 8 neurons; r^2^ = 0.292; ***p < 0.0001; dotted line indicates 95% confidence interval. (**c**) No correlation at I-Syns; n = 144 synapses from 8 neurons; r^2^ = 0.00005; NS; dotted line indicates 95% confidence interval. (**d**) Representative images of CaMKII enrichment and GluA1 labelling at DiSs before and after syn-cLTP; scale bar, 1 µm. (**e**) Correlation at PSD95 puncta at DiSs; n = 92 synapses from 8 neurons; r^2^ = 0.231; ***p < 0.0001; dotted line indicates 95% confidence interval. (**f**) No correlation at gephyrin puncta at DiSs; n = 93 synapses from 8 neurons; r^2^ = 0.012; NS; dotted line indicates 95% confidence interval.

We used the same neurons to test for synaptic CaMKII localization (in Fig. 2) and for GluA1 surface insertion (in Fig. 3) in response to syn-cLTP stimuli. This enabled us to plot the detected GluA1 surface insertion as a function of the CaMKII synaptic enrichment for each individual synaptic punctum. We found a clear and significant correlation between the enrichment of CaMKII and the signal intensity of newly-inserted GluA1 at SiSs, but not I-Syns (Fig. 4a–c and Supplementary Fig. Sc,d). At DiSs, a correlation was only observed for the PSD95 component; the apparent mild correlation at gephyrin puncta was not significant (Fig. 4d–f and Supplementary Fig. S4e,f). Overall, this further supports our findings that LTP can be induced at DiSs in response to a strong chemical LTP stimulus, and that the efficacy of LTP is correlated to the extent of CaMKII synaptic accumulation.

## Discussion

Dually innervated dendritic spines (DiSs; i.e. spines that are contain an inhibitory synapse in addition to the typical excitatory synapse) provide the potential for additional levels of signal computation, compared to both excitatory singly innervated spines (SiSs) and inhibitory synapse on dendritic shaft (I-Syns). The signal computation in DiSs remains largely unexplored, but a recent study showed that (i) excitatory LTP mechanisms are suppressed in DiSs and that (ii) this suppression can be overcome by blocking tonic inhibitory GABA_B_R signaling^3^. As an additional complementary computational mechanism, we showed here that (iii) the LTP suppression at DiSs can also be overcome by strong excitatory LTP stimuli. The LTP mechanisms elicited in DiS by strong LTP stimuli included CaMKII accumulation, spine growth, and surface insertion of AMPARs.

The GABAergic suppression of LTP mechanisms in DiSs is mediated by a reduction in the Ca^2+^ signal that is initiated by excitatory stimulation of NMDARs, and involves reduction in both the direct Ca^2+^ flux through NMDARs as well as the subsequent Ca^2+^ flux through voltage-dependent Ca^2+^ channels (VDCCs) that is triggered by NMDAR-mediated currents^3^. Based on this mechanism, LTP mechanism should be restored by increasing Ca^2+^ signal not only by preventing the GABA-mediated inhibition but also by directly increasing the excitatory stimulus, as we indeed observed here experimentally. Notably, as indicated above, the suppression of LTP mechanisms at DiSs appears to be specifically mediated by GABA_B_R signaling^3^, even though Ca^2+^ signals can be dampened by signaling through either GABA_A_Rs or GABA_B_Rs^3,5,31^.

Although we classify our excitatory LTP stimuli as “strong”, it should be noted that this could be said for any LTP-inducing stimulus (with “weak” stimuli instead either eliciting no effect or even the opposite, synaptic depression). Indeed, the previously used LTP stimuli by optical glutamate uncaging at individual dendritic spines were sufficiently strong to elicit LTP mechanisms at SiSs (and even at DiSs when combined with block of GABA_B_R signaling)^3^. Thus, these previously used stimuli are not weak either, even if they can be considered to be weaker when directly compared to the two stimuli that we utilized here. Our cLTP stimulus by bath application of glutamate is certainly stronger than the local glutamate uncaging; but even our syn-cLTP stimulus that relies on the stimulation of endogenous synaptic transmission should be considered to be stronger as it is longer lasting and involves global stimulation of many of the endogenous synapses, rather the local exogenous stimulation of individual spines that was used previously^3^.

In our hippocampal neurons, ~ 13% of all dendritic spines were DiSs, and these DiSs represented ~ 30% of all their inhibitory input. The corresponding numbers that were previously reported ranged from 4 to 14% and from 26 to 38%, respectively^4,19–22^. For example, the most recent of these papers reported that 10% of spines were DiS and ~ 35% of inhibitory synapses are on spines^22^. These numbers are for pyramidal neurons in cortex, however, they are similar to recent observations in hippocampus^3^. The variation in these numbers, although not excessive, might be explained by differences in previous experience of the analyzed animals. Indeed, whisker stimulation increased the number of DiS in the somatosensory cortex^19^. However, DiS formation does not appear to require functional glutamate or GABA release from the contacting excitatory or inhibitory inputs, at least in hippocampus^3^.

In response to LTP stimuli, CaMKII accumulated at SiSs but not at I-Syns, consistent with previous reports^17,24^. However, for the additional CaMKII accumulation at DiSs, the same accumulation was seen when measure as co-localization with the excitatory synapse marker PSD95 or with the inhibitory synapse marker gephyrin. This may be attributed at least in part to the resolution limits of light microscopy. Indeed, the PSD95 and gephyrin puncta within DiSs are at less than a 500 nm distance away from each other (measured from their center, as defined by our analysis method), placing even their maximal distance only barely over resolution limit of light microscopy of > 200 nm. However, for the surface insertion of GluA1 at DiSs, significantly more GluA1 insertion was detected associated with the PSD95 puncta compared to the gephyrin puncta. This distinction may reflect the surface accumulation of GluA1 directly within the structure of excitatory synapses, compared to CaMKII accumulation that includes also more peripheral regions, as indeed previously suggested by electron-microscopy^32^. Additionally, the curvature of spines would place staining of specific spine structures on their outside (as for surface GluA1) further apart compared to their staining on the inside (as for intracellular CaMKII).

Overall, DiSs occur more frequently than commonly recognized, and our understanding about their physiological and pathological functions in neurons remains sparse^5,19,33^. Similarly, there is still limited knowledge about the obvious potential of DiSs for additional computation by facilitated cross-talk between the excitatory and inhibitory inputs^3,5,31^. Much remains to be learned, but the current study unveiled an interesting additional aspect: LTP-mechanisms at DiSs can be “de-suppressed” not only by reducing inhibitory signals, but also by increased excitatory LTP stimuli.

## Methods

### Experimental model and subject details

All animal treatment was approved by the University of Colorado Institutional Animal Care and Use Committee and conducted according to the guidelines by the American Veterinary Medical Association. Animals are housed at the Animal Resource Center at the University of Colorado Anschutz Medical Campus (Aurora, CO) and are regularly monitored with respect to general health, cage changes, and overcrowding. Pregnant Sprague–Dawley rats were supplied by Charles River Labs. Rat pups were euthanized on postnatal day P0 using scissors for rapid decapitation to prepare rat hippocampal cultures that were imaged on day in vitro (DIV) 17–20.

### Material and DNA constructs

Material was obtained from Sigma, unless noted otherwise. The fluorescently-labelled intrabodies against PSD95 and gephyrin have been characterized to not affect synaptic functions^17,18^; successful co-labeling with an intrabody against CaMKII has been described previously^14,17^. Expression of the intrabodies are driven by the CAG promoter. All constructs were validated by sequencing. The pairs of PSD95 and gephyrin intrabodies were tagged with mCherry and mTurquoise (in Fig. 1) or with mTurquoise and mScarlet (in Figs. 2, 3 and 4), as also indicated in figure legends; the CaMKII intrabody was tagged with YFP2 in all cases.

### Primary hippocampal culture preparation

To prepare primary hippocampal neurons, hippocampi were dissected from mixed sex rat pups (P0), dissociated in papain for 1 h, and plated at 100,000 cells/mL on 18 mm No. 1 deckgläser cover glasses in plating media (MEM containing 10% FBS, 1% Penicillin–Streptomycin). On DIV 1 plating media was replaced with feeding media (Neurobasal A containing 2% B27 and 1% Glutamax). On DIV 7, half of conditioned feeding media was replaced with fresh feeding media containing 2% 5-Fluoro-2’-deoxyuridine (FdU).

### Image acquisition

Neurons were imaged using a Axio Observer microscope (Carl Zeiss) fitted with a 63 × Plan-Apo/1.4 numerical aperture (NA) objective, 50 μm pinhole, using 445, 515, 567 and 647 nm laser excitation and a CSU-XI spinning disk confocal scan head (Yokogawa) coupled to an Evolve 512 EM-CCD camera (Photometrics) and controlled using Slidebook 6.0 software (Intelligent Imaging Innovations [3i]. The 63 × immersion objective (4.92 pixels/micron) was used to acquire all images.

DIV 15–18 rat neuronal cultures were transfected with Lipofectamine 2000 (Invitrogen) express intrabodies and cell fill and imaged 24–48 h after (see figure legends for construct details). For experiments measuring dendritic spine size, Halotag was transfected and labelled with 100 nM Janelia Fluor Halotag ligand-646 for 10 min prior to imaging. Images were collected at 32 °C in HEPES buffered imaging solution (ACSF) containing (in mM) 130 NaCl, 5 KCl, 10 HEPES pH 7.4, 20 Glucose, 2 CaCl_2_, 1 MgCl_2_.

### Chemical LTP stimulation

In the imaging chamber, glutamate-induced chemical LTP (cLTP) was induced with 100 μM glutamate and 10 μM glycine for 45 s. Treatments were followed by washout with 5 volumes (5 mL, 0.1 mL/s of fresh ACSF. Images were captured 5 min after washout.

For glycine-stimulated chemical LTP (syn-cLTP), the baseline solution of ACSF contained 1 mM TTX and was incubated for 1 h prior to stimulation. To stimulate cells, this baseline solution was exchanged for modified ACSF containing 0 mM MgCl_2_ and 200 µM glycine for 5 min. Cells were then observed for up to 20 min in baseline ACSF without TTX prior to image capture. Mock conditions replaced baseline + TTX with ACSF containing 1 mM Mg^2+^ and 0 mM glycine.

### GluA1 labeling and application

An antibody directed against the extracellular N-terminal domain of GluA1^25,28,30^ was affinity purified and conjugated to Alexa Fluor 647 dye using the Molecular Probes Alexa Fluor 647 Antibody Labeling Kit (Invitrogen, cat. no. A20186). Briefly, 100 µL of a 1.2 mg/mL anti-GluA1 antibody solution (containing 0.1 M sodium bicarbonate) was incubated with Alexa 647 Fluor reactive dye for 30 min at room temperature with gentle rocking. Alexa 647-conjugated anti-GluA1 antibody was separated from unbound dye by gel-filtration chromatography. The protein concentration after labeling was 0.6 mg/mL (in PBS, pH 7.2, 2 mM sodium azide) with 2.1 mol of dye per mole of protein. Labeled antibody was aliquoted and stored at -20 °C.

For live GluA1 detection in syn-cLTP experiments, cultures were incubated with unlabeled GluA1 antibody (1:300) in baseline + TTX condition to block existing surface receptors for 1 h. Then, neurons were exposed to either ACSF (vehicle control) or syn-cLTP for 5 min, and subsequently washed with 5 volumes of ACSF over 1 min. Neurons were then treated with Alexa 647-conjugated GluA1 antibody (1:300) to label newly inserted surface receptors. After 20 min, neurons were again washed with 5 volumes of ACSF to remove unbound GluA1-647 antibody and imaged.

### Analysis of CaMKII synaptic enrichment and GluA1 surface expression

Images of individual neurons from at least two independent cultures were acquired by 0.5 μm steps over 6 μm. 2D maximum intensity projection images were then generated and analyzed using ImageJ.

Threshold for entire cell was generated by gaussian blur followed by Otsu threshold of the cell fill fluorescence. Thresholds for PSD95 and gephyrin objects were experimentally defined using fluorescence intensity-based masking. To generate a mask that included only dendritic shaft while omitting either excitatory and inhibitory synapses, PSD-95 and gephyrin-containing synaptic regions, the synapse masks was dilated and subtracted from the cell fill mask. Individual synapses were identified using the “Analyze Particles” function of ImageJ. For each synapse type, distance to the nearest neighbor of the opposite type of synapse was calculated. DiSs were identified by PSD95 puncta or gephyrin puncta with proximal gephyrin or PSD95 labeling within 0.5 µm, respectively (measured as center-to-center distance between objects). SiSs were identified by PSD95 puncta that lack such a proximal gephyrin marker within 0.75 µm. I-Syns were identified by gephyrin puncta that lack such a proximal PSD95 marker within 0.75 µm. Puncta with nearest neighbors within the intermediate range of 0.5–0.75 µm were excluded from the analysis, as their identity as SiS, I-Syn or DiS is less clear. CaMKII synaptic enrichment values were calculated by mean fluorescence intensity value within each mask type normalized to the mean intensity value within the dendritic shaft mask.

### Spine growth measurements

Images were background subtracted and ROIs were randomly drawn over dendritic spines based on cell fill fluorescence of Halo647. Identification of dual synapses was made by inclusion of gephyrin puncta. Integrated fluorescent intensity of Halo647 was utilized to measure changes in spine size within stable ROI before and for 20 min after the syn-cLTP stimulus.

### Quantification and statistical analysis

All data are shown as mean ± SEM. Statistical significance is indicated in the figure legends. Statistics were performed using Prism (GraphPad) software. Imaging experiments were obtained and analyzed using SlideBook 6.0 software. All comparisons between two groups met parametric criteria, and independent samples were analyzed using unpaired, two-tailed Student’s *t*-tests. Comparisons between three or more groups meeting parametric criteria were done by one-way ANOVA with specific post-hoc analysis indicated in figure legends. Welch’s ANOVA was chosen due to its robustness against violations of the assumption of equal variances, ensuring reliable comparisons even when heterogeneity of variances exists across groups. For the paired analysis per neuron, repeated measure two-way ANOVA was used with Bonferroni multiple comparison post-hoc analysis. Asterisks represent level of significance: *p < 0.05; **p < 0.01; ***p < 0.001, ****p < 0.0001.

### Supplementary Information

Supplementary Figures.

## Data Availability

The datasets generated during this study are available through Mendeley (https://data.mendeley.com/datasets/k3zcvkzvtp).
