# Supplementary material for: Induction of LTP mechanisms in dually innervated dendritic spines
Source: Sci Rep. 2024 Jul 9;14:15855. doi: 10.1038/s41598-024-66871-8 (PMC11233660; doi:10.1038/s41598-024-66871-8)
Supplement: Supplementary file 1 — Supplementary Figures. [file 41598_2024_66871_MOESM1_ESM.pdf]

Induction of LTP mechanisms in dually innervated dendritic spines by strong excitatory stimuli

Jonathan E. Tullis[1], and K. Ulrich Bayer[1,2*]

[1]Department of Pharmacology, and [2]Program in Neuroscience, University of Colorado Anschutz Medical Campus, Aurora, CO 80045, USA

*Corresponding author. Email: ulli.bayer@cuanschutz.edu.

Supplementary Figures S1-S4

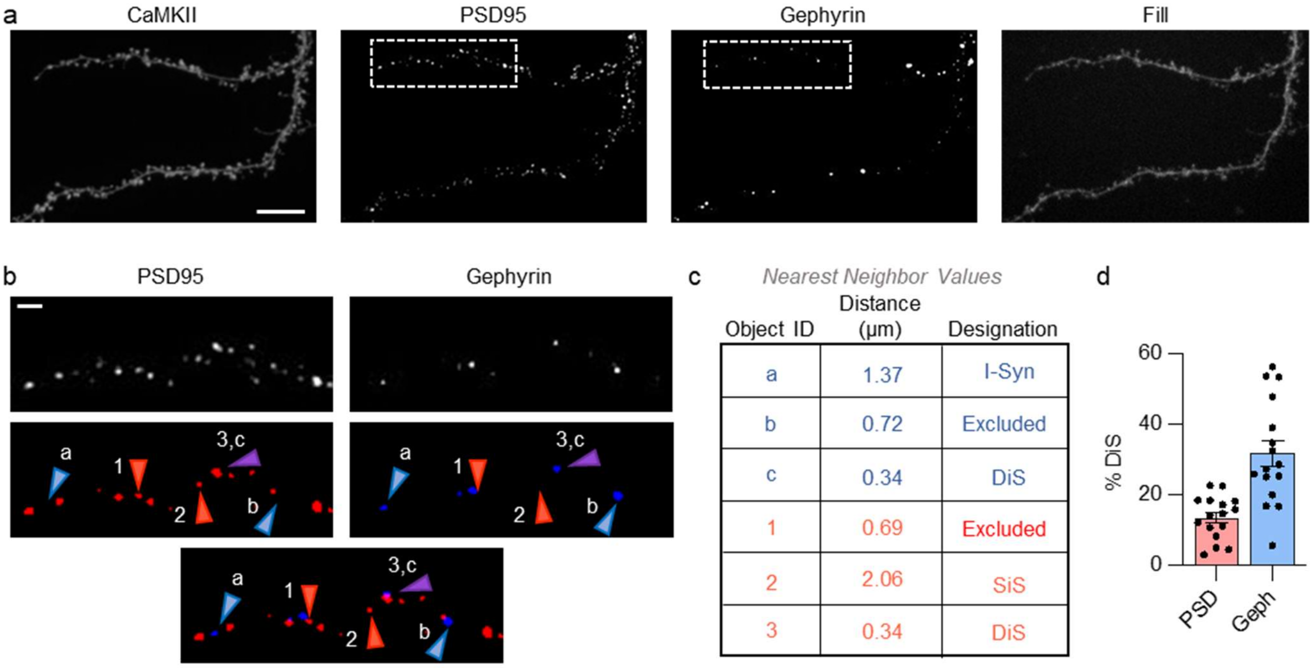

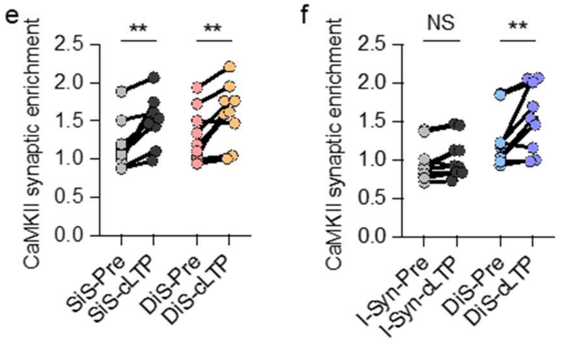

Supplementary Figure S1. Identification of dual synapses.

(**a**) Representative image of CaMKII, PSD95, and gephyrin intrabody, along with an iRFP cell fill expressed in cultured hippocampal neurons; scale bar, 10 µm. ImageJ analysis-generated masks based on the signal for CaMKII, PSD95, gephyrin and cell fill.

(**b**) Subsection of dendrite from inset outlined in the image of the PSD95 mask in panel A. Masked image and arrows designating PSD95 (red) and gephyrin objects (blue). Purple arrows represent objects at DiS; scale bar, 2 µm.

(**c**) Table of masked objects from panel B and the distance to nearest neighbor of the opposite synapse type. Designation of synapse subtype based on nearest neighbor also shown. Synapses within intermediate distances of 0.5-0.75 µm were excluded from quantification.

(**d**) Percentage of DiS for both PSD95 and gephyrin objects; n = 16. Mean +/- SEM shown.

(**e**) Data from Fig. 1b is shown here *per neuron*. cLTP increased synaptic enrichment of CaMKII within PSD95 puncta at both SiSs and DiSs (N = 7 neurons; $^{**}p<0.0001$ in Bonferroni multiple comparisons test after two-way ANOVA). The initial two-way ANOVA examined the effect of synapse type and synaptic stimulation on CaMKII synaptic enrichment values. The results are not indicated, but there was a statistically significant effect of cLTP ($F(1, 16) = 32.64$; $p < 0.0001$) but not synapse type ($F(1,16) = 0.52$; $p = 0.48$) on CaMKII synaptic enrichment values.

(**f**) Data from Fig. 1c is shown here *per neuron*. cLTP increased synaptic enrichment of CaMKII within gephyrin puncta at DiSs but not I-Syns (N = 7 neurons; $^{**}p<0.01$ in Bonferroni multiple comparisons test after two-way ANOVA). The initial two-way ANOVA examined the effect of synapse type and synaptic stimulation on CaMKII synaptic enrichment values. The results are not indicated, but there was a statistically significant effect of synaptic stimulation ($F(1,16) = 11.71$; $p = .0035$) and synapse type ($F(1,16) = 6.92$; $p = 0.018$) on CaMKII synaptic enrichment values.

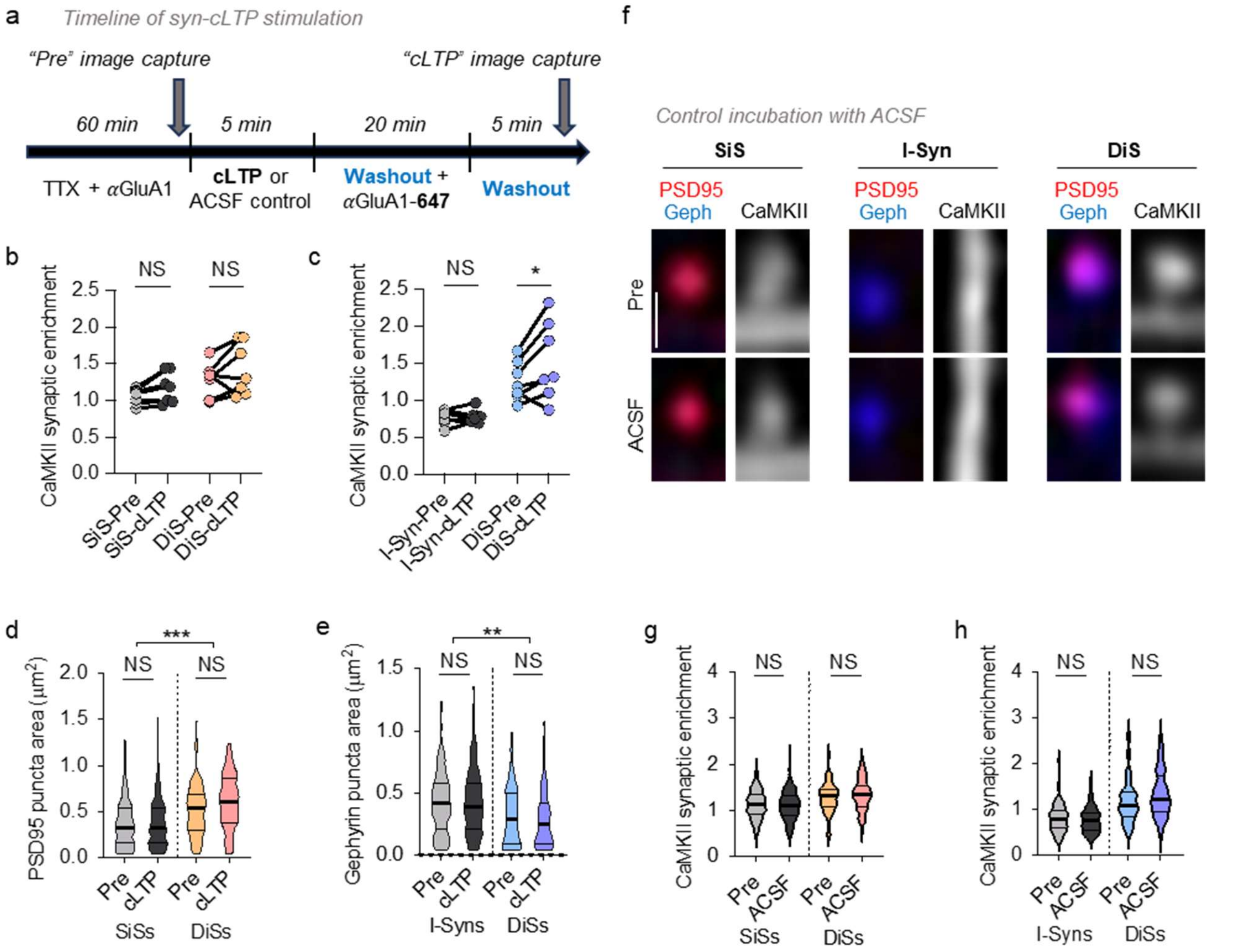

Supplementary Figure S2. Synaptic chemical LTP (syn-cLTP) induction method and LTP readout. (**a**) Schematic diagram of the timeline of the live imaging methodology to induce LTP through synaptic release of glutamate, including for measurement of surface expression of new GluA1. First, cells were incubated in 1 μM TTX with an unlabeled GluA1 antibody for 60 min, to reduce activity and block existing surface GluA1. The "pre" image was captured at the end of this incubation. Immediately after the first image capture, the imaging ACSF was exchanged for 5 min incubation in Mg^{2+}-free ACSF with 200 μM glycine for syn-cLTP induction, followed by washout with standard ACSF for 20 min containing Alexa 647-conjugated GluA1 antibody to detect newly inserted receptor subunits. Finally, the unbound GluA1-647 antibody is washed out before capture of the second image (cLTP image).

(**b**) Data from Fig. 2b is shown here *per neuron*. Post-hoc analysis did not indicate significant synaptic accumulation of CaMKII within PSD95 puncta at both SiSs and DiSs (N = 7 neurons; NS in Bonferroni multiple comparisons test after two-way ANOVA), unlike in the analysis per synapse. However, the overall effect of syn-cLTP did show an effect in the initial two-way ANOVA that examined the effect of synapse type and synaptic stimulation on CaMKII synaptic enrichment values. The results are not indicated, but there was a statistically significant effect of synaptic stimulation ($F(1, 12) = 5.62$; $p = .035$ but not synapse type ($F(1,12) = 4.13$; $p = 0.065$) on CaMKII synaptic enrichment values.

(**c**) Data from Fig. 1c is shown here *per neuron*. Syn-cLTP increased synaptic enrichment of CaMKII within gephyrin puncta at DiSs but not I-Syns (N = 7 neurons; $*p<0.05$ in Bonferroni's multiple comparisons test after two-way ANOVA). The initial two-way ANOVA examined the effect of synapse type and synaptic stimulation on CaMKII synaptic enrichment values. The results are not indicated, but

there was a statistically significant effect of synaptic stimulation ($F(1, 12) = 5.74$; $p = .034$) and synapse type ($F(1,12) = 16.64$; $p = 0.0015$) on CaMKII synaptic enrichment values.

(**d**) Syn-cLTP had no effect on PSD95 object area. PSD95 objects at DiSs are significantly larger than at SiSs; n = 712, 683, 76, 92 synapses from 7 neurons; ***$p<0.001$; One-way ANOVA with Dunnett's multiple comparisons test.

(**e**) Syn-cLTP had no effect on Gephyrin object area. Gephyrin objects at DiSs are significantly smaller than at I-Syns; n = 148, 144, 76, 93 synapses from 7 neurons; **$p<0.01$; One-way ANOVA with Dunnett's multiple comparisons test.

(**f**) Representative images of CaMKII localizing to SiS, I-Syn, and DiS before and after ACSF control; scale bar, 1 μm.

(**g**) ACSF control had no effect on synaptic enrichment of CaMKII at PSD95 puncta at either SiSs or DiSs; n = 449, 453, 54, 59 synapses from 6 neurons; NS; One-way ANOVA with Dunnett's multiple comparisons test.

(**h**) ACSF control had no effect on synaptic enrichment of CaMKII at gephyrin puncta at either SiISs or DiSs; n = 103, 99, 55, 59 synapses from 6 neurons; NS; One-way ANOVA with Dunnett's multiple comparisons test.

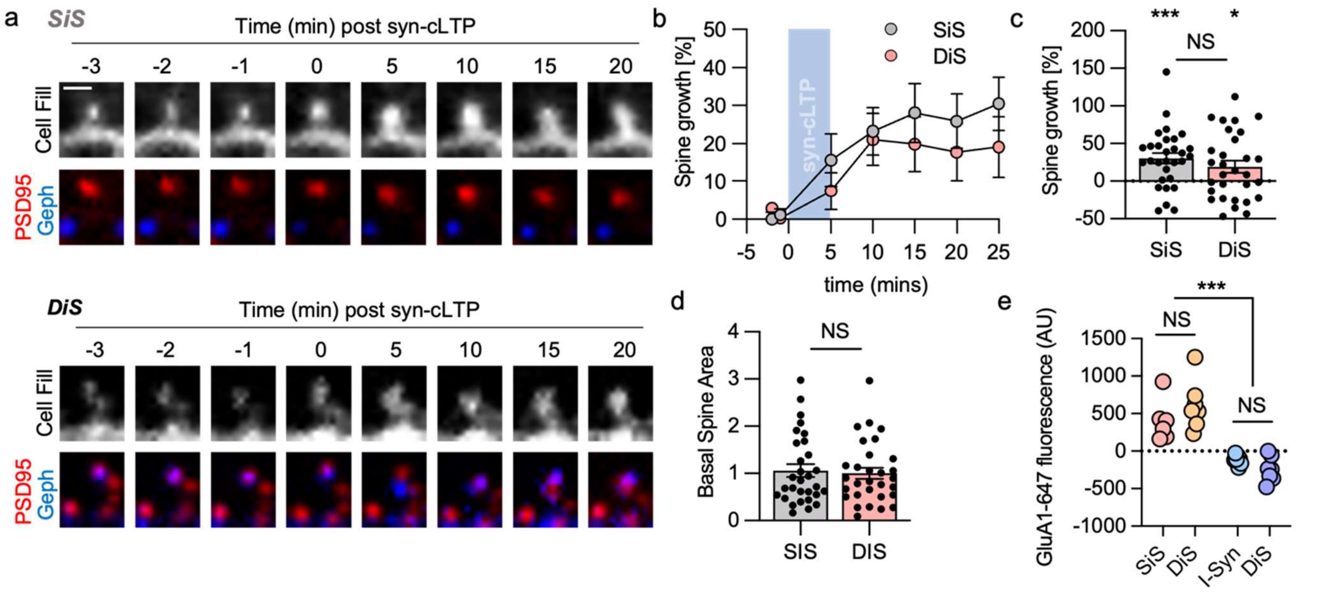

Supplementary Figure S3. Structural LTP at DiSs measured by dendritic spine growth.

(**a**) Representative images of structural LTP observed after syn-cLTP for both SiS (top) and DiS (bottom); scale bar, 1 µm.

(**b**) Timecourse of spine growth measured as change in spine head area for DiSs and SiSs. The 5 minute syn-cLTP treatment is indicated by blue shadow on timecourse.

(**c**) Spine growth was observed in both SiSs and DiSs at 20 min after syn-cLTP; n = 30 spines (from 4 different neurons); $*p<0.05$, $***p<0.001$; one-sample t-test. The spine growth did not significantly differ between the two synapse types; NS, unpaired two-tailed student's t-test.

(**d**) Basal spine area was not significantly different between the two spine populations; n = 4 neurons (7-8 spines analyzed per cell); NS; unpaired two-tailed student's t-test.

(**e**) Syn-cLTP increased GluA1 surface expression within PSD95 puncta at SiSs and DiSs (measured after blocking the pre-existing surface GluA1 with unlabeled antibody), but significantly less within gephyrin puncta at either SiISs or DiSs as in Fig. 3b, but here shown *per neuron*; n = 7; $***p<0.001$; Welch's one-way ANOVA with Dunnett's multiple comparisons test.

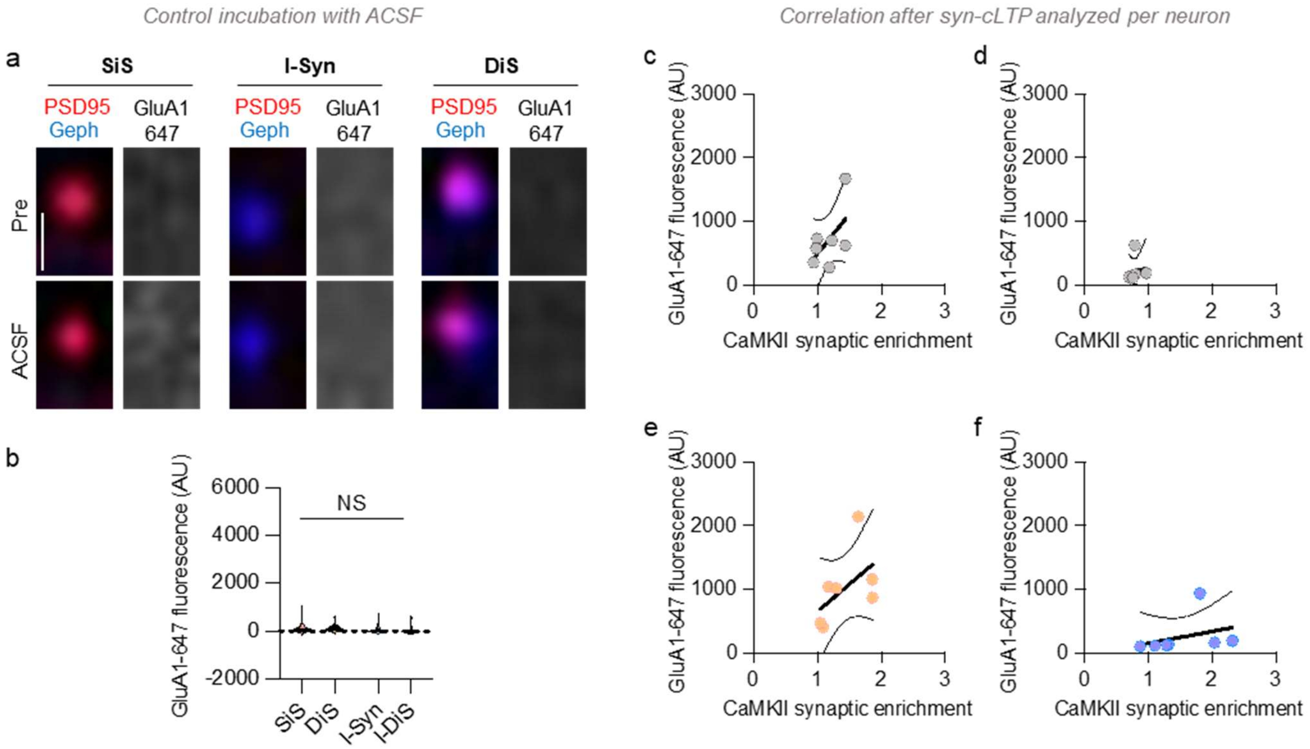

Supplementary Figure S4. ACSF control does not induce increased GluA1 surface expression.
(**a**) Representative images of GluA1-647 at SiS, I-Syn, and DiS before and after ACSF control; scale bar, 1 µm.
(**b**) The ACSF control treatment does not increase GluA1 surface expression within PSD95 puncta at SiSs or DiSs, nor within gephyrin puncta at either I-Syns or DiSs; n = 452, 59, 132, 73 synapses from 6 neurons; NS; One-way ANOVA with Dunnett's multiple comparisons test.
(**c-f**) Linear regression analysis of GluA1 surface expression as a function of CaMKII enrichment (in response to syn-cLTP) as in Fig. 4b,c,e,f, but here shown *per neuron (n=8)* instead of per synapse. SiSs; r^2 = 0.33; I-Syns; r^2 = 0.09; PSD-DiSs; r^2 = 0.28; Gephyrin-DiSs; r^2 = 0.11.
